# Trends of incidence and prognosis of upper tract urothelial carcinoma

**DOI:** 10.17305/bjbms.2020.5345

**Published:** 2021-10

**Authors:** Jianping Wu, Shuqiu Chen, Xiaoli Wu, Weipu Mao, Yali Wang, Bin Xu, Donghui Zheng, Ming Chen

**Affiliations:** 1 Department of Urology, Affiliated Zhongda Hospital of Southeast University, Nanjing, Jiangsu province, China; 2 Department of Obstetrics and Gynecology, Affiliated Nanjing Maternity and Child Health Hospital, Nanjing Medical University, Nanjing, Jiangsu province, China; 3 Surgical Research Center, Institute of Urology, Southeast University Medical School, Nanjing, China; 4 Department of Urology, Nanjing Lishui District People’s Hospital, Zhongda Hospital Lishui Branch, Southeast University, Nanjing, China; 5 Department of Nephrology, Affiliated Huai’an Hospital of Xuzhou Medical University, Huai’an, Jiangsu province, China

**Keywords:** Upper tract urothelial carcinoma, incidence, prognostic nomogram, survival outcome, SEER

## Abstract

The purpose of this study was to investigate trends in the incidence of upper tract urothelial carcinoma (UTUC) in patients and to establish a reliable and practical nomogram based on significant clinical factors to predict the overall survival (OS) and cancer-specific survival (CSS) of UTUC patients. The Surveillance, Epidemiology, and End Results (SEER) database was used to extract data on UTUC patients between 1988 and 2015. Incidence was calculated using Joinpoint regression software, and trends were quantified by annual percentage change (APC). A nomogram was constructed using R software to predict the OS and CSS probabilities for individual patients. From 1988 to 2015, the incidence of UTUC showed a downward trend (1988: 1.57/100,000 to 2015: 1.51/100,000; APC=-0.1). After stratification according to sex, age and primary site, we found that the incidences of UTUC in males, patients 70+ years old and the renal pelvis were higher than those in females, patients <70 years old and ureter cancer patients. In the training cohort, the nomogram established based on multivariate Cox regression results showed better OS and CSS accuracy (OS: C-index=0.701, AUC=0.736; CSS: C-index=0.729, and AUC=0.688) than SEER stage. In addition, the calibration curves showed good consistency between the predicted and actual 3-, 5- and 10-year OS and CSS rates of the nomogram. In the past 30 years, the incidence of UTUC has shown a general downward trend, and the prognostic nomogram we established can provide a personalized risk assessment for the survival of UTUC patients.

## INTRODUCTION

Upper tract urothelial carcinoma (UTUC) is a relatively rare tumor, including carcinoma of the renal pelvis and carcinoma of the ureter, which account for approximately 5%-10% of urothelial carcinomas (UCs).[1, 2] In the United States, there were approximately 15,000 newly diagnosed cases of UTUC in 2014.[3] UTUC has the characteristics of multicentric tumor growth and urinary dissemination tendency and has a higher grade and stage at the time of diagnosis.[4] The 5-year cancer-specific survival rate of UTUC patients is 50-80%.[5]

The incidence of UTUC is high in Taiwan, especially on the southwest coast of the island, which accounts for 20-25% of UC in the region.[6, 7] Some epidemiological studies have shown that the annual incidence rate in Western countries is approximately 2 cases per 100,000 residents.[8] The majority of UTUC patients in Europe and the United States are male, with a male to female ratio of approximately 2:1, it is more common in individuals aged 70-90 years, and the incidence of renal pelvic cancer is approximately twice that of ureter cancer.[9] However, to the best of our knowledge, it is unclear what trends in UTUC have changed over the past three decades and whether any changes have been driven by factors such as age, sex and primary site.

The American Joint Committee on Cancer (AJCC) tumor node metastasis (TNM) staging system is widely used to evaluate the prognosis of patients with UTUC.[10] However, other factors, such as age, sex, race, marital status, SEER stage, grade and treatment pattern, can also affect the outcome of UTUC patients. Therefore, it was necessary to establish a comprehensive prognostic assessment system, including clinicopathological and demographic variables, which may be used in clinical practice. The nomogram was based on the equations derived from the regression coefficients of each variable and integrates many prognostic factors, which can more accurately predict the individual survival of patients with UTUC.[11]

The purpose of the current study was to estimate the incidence of UTUC based on age, sex and primary site and to establish a reliable and practical nomogram based on significant clinical factors to predict the overall survival (OS) and cancer-specific survival (CSS) of UTUC patients. Therefore, this study used the Surveillance, Epidemiology, and End Results (SEER) database to study the incidence trends and to establish a prognostic nomogram in UTUC patients from 1988 to 2015. We hope that the results of this study can improve the OS and CSS rates of patients with UTUC.

## MATERIALS AND METHODS

### Patients selection

The data used in our study were retrieved from the National Cancer Institute-funded SEER database. The SEER database covers approximately 28% of the US population and includes demographic information and cancer characteristics, such as year of diagnosis, diagnosis age, sex, race, marital status, primary tumor site, tumor grade and stage, histological type, tumor-node-metastasis (TNM) stage, treatment modality and survival time.[12]

The study was conducted in accordance with the Declaration of Helsinki (as revised in 2013). This study used previously collected deidentified data, which were deemed exempt from review by the Ethics Committee of the Affiliated Zhongda Hospital of Southeast University.

### Estimate trends of incidence of UTUC patients

We used SEER*Stat software version 8.3.6 (https://seer.cancer.gov/seerstat/) to collect incidence information (Incidence - SEER 9 Regs Research Data, Nov 2018 Sub (1975-2016) < Katrina/Rita Population Adjustment). *The International Classification of Diseases for Oncology, Third Edition* (ICD-O-3) site codes C65.9-renal pelvis and C66.9-ureter were used to identify patients diagnosed with UTUC between 1988 and 2015. We divided all patients into males and females based on sex. According to the age at diagnosis, the population was divided into three groups: the <60 years group, the 60-69 years group and the 70+ years group. On the basis of the primary site, the patients were divided into renal pelvis cancer and ureter cancer.

### Analysis of the survival trends of UTUC patients

The National Cancer Institute’s SEER*Stat software version 8.3.6 (https://seer.cancer.gov/seerstat/) (SEER 18 Regs Custom Data (with additional treatment fields), Nov 2018 Sub (1975-2016 varying) database) was used to analyze the survival trends of UTUC patients. The exclusion criteria were as follows: (a) unknown marital status or domestic partner (n=990); (b) age < 18 years (n=7); (c) unknown survival time (n=72); (d) two or more primary tumors (n=12,710); and (e) unknown surgery (n=32). Finally, we were left with 10,852 eligible patients diagnosed with UTUC.

Variable definitions of information about age at diagnosis, sex, marital status, race, origin, primary site, histological type, grade, SEER stage, surgery, radiotherapy, chemotherapy, cause of death and survival time can be found in the SEER database. The starting point of the follow-up was the date of diagnosis of UTUC, and the end point was cancer-specific death or the last follow-up as of December 2016. The overall survival (OS) time corresponded to the length of time from the date of diagnosis to death from any cause or the date on which data were censored. When analyzing cancer-specific survival (CSS), mortality cases associated with other causes were excluded.

### Statistical analysis

The incidence calculations were performed per 100,000 people and adjusted by age to the 2000 U.S. standard population. To compare the incidence trends of UTUC from 1988 to 2015 by stage, we performed regression analysis using Joinpoint regression software from the National Cancer Institute’s SEER program, which detects trends in incidence and can determine the start and end years in which incidence increases and/or decreases. The regression model is used to estimate the rates between the two years from the analysis in Joinpoint, the annual percentage change (APC) of the rates and the 95% confidence interval (CI).

R software was used to randomize all patients into a training and validation set at a ratio of 2:1. Univariate and multivariate Cox regression models were performed to estimate the hazard ratios (HRs) and 95% CIs to analyze independent prognostic factors of UTUC OS and CSS in the training set. We constructed a nomogram based on the multivariate Cox regression results. Receiver operating characteristic (ROC) curves were used to assess the predictive ability of the nomograms and to determine the area under the curve (AUC). In addition, by comparing the predicted survival time with the observed survival time, the predictive performance of the nomogram was evaluated using the consistency index (C-index) and calibration curve, and the nomogram was calibrated for 3-, 5- and 10-year OS and CSS. The C index was similar to the AUC but seems to be more suitable for censored data. The value of the C-index statistic was between 0.5 (nondiscrimination) and 1 (perfect discrimination), and a higher C-index value indicates a better prognostic model. These evaluations were performed using bootstrapping with 1000 resamples. There was no direct clinical interpretation of the C-index. Therefore, we also analyzed the decision curve analysis (DCA), which is a novel method to evaluate the predictive model for evaluating net benefits from the perspective of clinical outcome.

SEER*Stat software version 8.3.6 (National Cancer Institute), Joinpoint regression software version 4.5.0.0 (Statistical Methodology and Applications Branch, Surveillance Research Program, National Cancer Institute) and the statistical software package R version 3.5.3 (http://www.r-project.org/) were used to calculate age-adjusted incidence. *P*-value ≤ 0.05 (two-sided) was considered significant.

## RESULTS

### Incidence trends of UTUC patients

**FIGURE 1:**
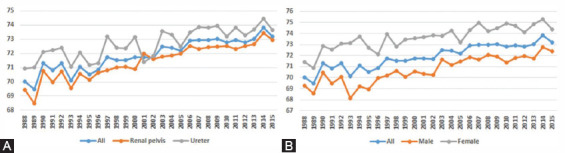
Change in average age at onset of UTUC patients from 1988 to 2015. A. Primary site; B. Sex.

From 1988 to 2015, the average age at onset of UTUC increased from 70.01 years in 1988 to 73.20 years in 2015, and the mean age of onset of UTUC in females and the ureter was higher than that in males and the renal pelvis (Figure 1).

**FIGURE 2:**
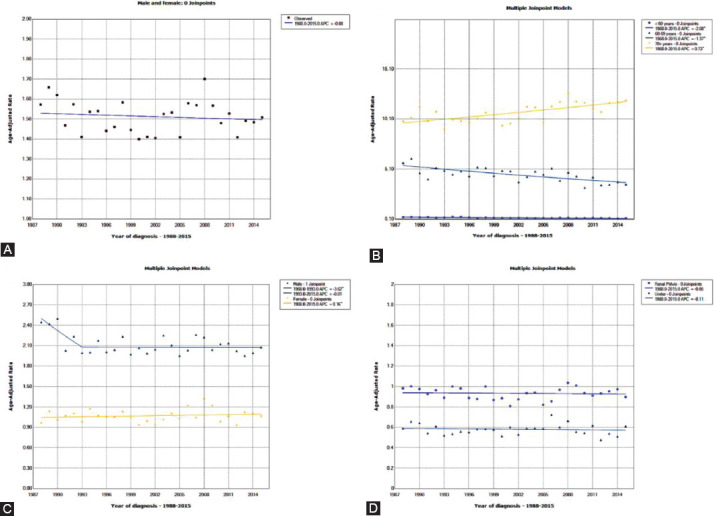
Change in the incidence of UTUC patients from 1988 to 2015. A. All patients; B. Grouped by age; C. Grouped by sex; D. Grouped by primary site.

The incidence of UTUC showed a downward trend (1988: 1.57/100,000 persons to 2015: 1.51/100,000 persons) between 1988 and 2015 (Figure 2A). The average APC of age-adjusted incidence among all UTUC patients was -0.1 (95% CI: -0.2 to 0.0). In the same period, the incidence of patients aged 70+ was significantly higher than that of patients <60 years and 60-69 years (Figure 2B). The incidence of UTUC patients aged <60 years (APC=-2.1, 95% CI: -2.3 to -1.9) and 60-69 years (APC=-1.4, 95% CI: -1.5 to -1.2) was decreasing, while the incidence of 70+ years patients was increasing (APC=0.7, 95% CI: 0.6 to 0.8) (Table 1). After stratification according to sex and primary site, we found that the incidences of UTUC in males and in the renal pelvis were higher than those in females and the ureter (Figure 2C, D).

**TABLE 1:**
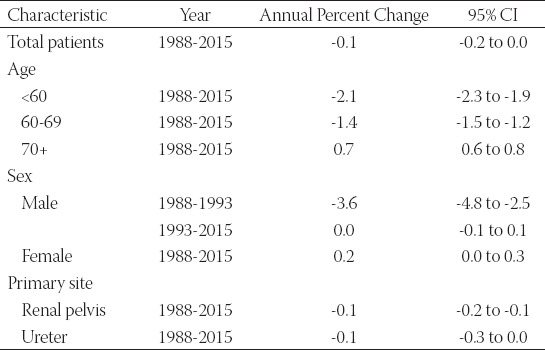
Changes in the incidence of upper tract urothelial carcinoma (UTUC) from 1988 to 2015

**FIGURE S1:**
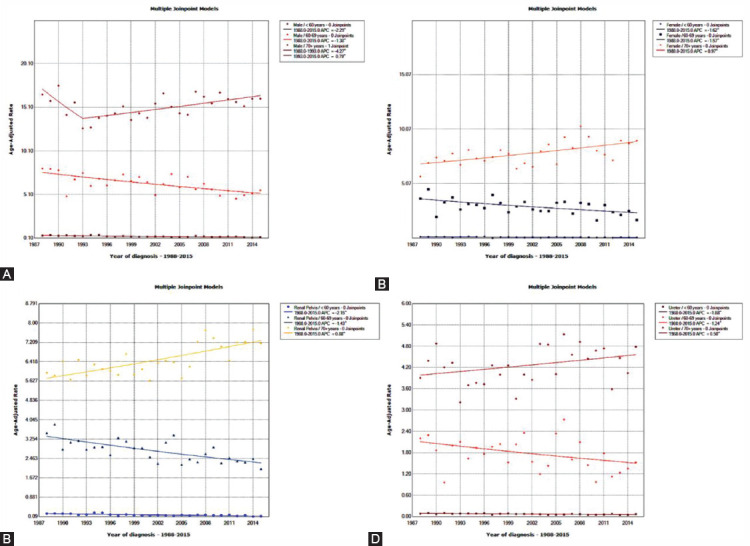
Stratified analysis of changes in the incidence of UTUC patients from 1988 to 2015. A. Grouped by sex and age; B. Grouped by primary site and age.

Subsequently, we stratified patients by sex and primary site to detect the effect of age on incidence (Supplementary Table 1). Among the four groups of 70+ years, only the male 70+ years group experienced a decrease in incidence from 1988 to 1993 (Supplementary Figure 1A), while the remaining three groups of 70+ years showed an increasing trend. In both males and females, in the renal pelvis and ureter, the incidence of UTUC in patients <60 years and 60-69 years old showed a decreasing trend (Supplementary Figure 1B-D).

### Demographic and clinicopathologic characteristics

From 1988 to 2015, our study cohort included 10,852 eligible UTUC patients, including 7,234 patients in the training cohort and 3,618 patients in the validation cohort. Table 2 shows the demographic and clinical characteristics of patients with UTUC. In the whole cohort, patients were more likely to be older (70+ years: 61.2%), white (86.1%), non-Spanish-Hispanic-Latino (92.2%), and married (58.2%). Tumors more frequently originated in the renal pelvis (66.3%), with the most common types being transitional cell carcinoma (92.1%), regional (50.8%), and high grade (grade III-IV: 61.2%). In addition, the majority of patients underwent surgical treatment (78.4%).

**TABLE 2:**
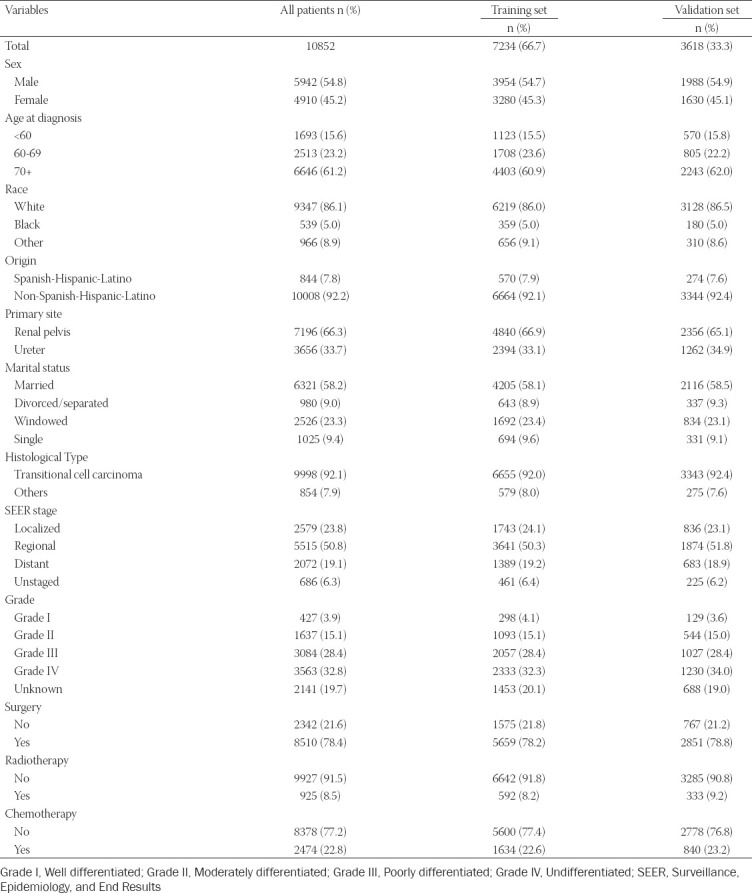
Baseline demographic and clinical characteristics

### Identification of prognostic factors of OS and CSS in the training set

In the training set, univariate and multivariate Cox regression were used to analyze the factors related to OS and CSS in patients with UTUC (Table 3). Univariate Cox regression analysis showed that sex, age at diagnosis, marital status, histological type, SEER stage, grade, surgery, radiotherapy and chemotherapy were related factors of OS and CSS of UTUC patients. Incorporating the univariate Cox regression correlates into multivariate Cox regression analysis, we found that sex, age at diagnosis, marital status, histological type, SEER stage, grade, surgery, radiotherapy and chemotherapy were independent risk factors for OS, and age at diagnosis, primary site, marital status, histological type, SEER stage, grade, surgery, radiotherapy and chemotherapy were independent risk factors for CSS.

**TABLE 3:**
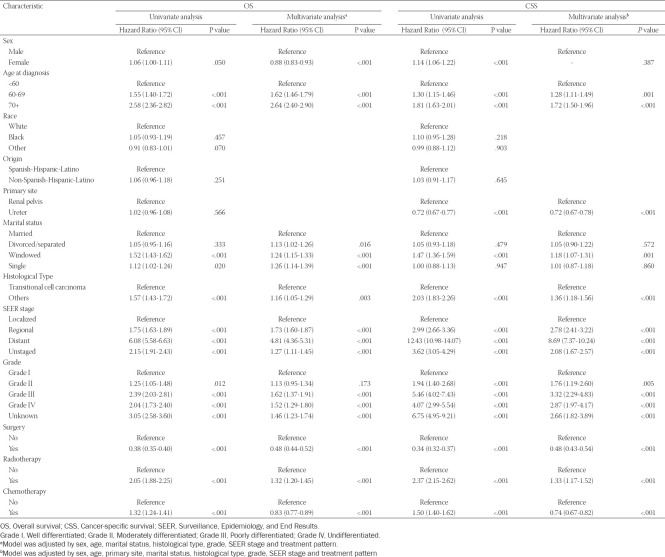
Univariate and multivariate analysis of overall survival (OS) and cancer-specific survival (CSS) rates in the training set

### Prognostic nomograms for OS and CSS

**FIGURE 3:**
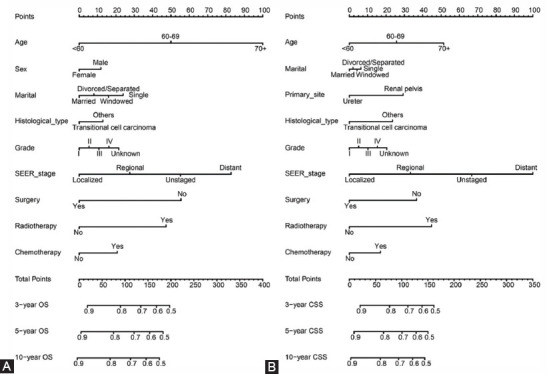
Nomogram predicting 3-, 5-, and 10-year overall survival (OS) and cancer-specific survival (CSS) rate of UTUC patients. A. OS rate; B. CSS rate.

Based on the multivariate Cox regression analysis results, we constructed 3-, 5- and 10-year OS and CSS prognostic nomograms for UTUC patients (Figure 3). Each subtype of the variables on the nomogram corresponds to a point on the “Point” scale. By adding the scores associated with each variable and projecting the “Total point” to the lowest number, the probabilities of OS and CSS for 3, 5, and 10 years can be estimated.

The length of the line corresponding to each variable in the nomogram represents the influence of the predictive variable on the survival outcome. We found that age contributed the most to survival outcome in the OS nomogram, while SEER stage contributed the most to the survival outcome in the CSS nomogram.

### Nomograms validation and calibration

**FIGURE 4:**
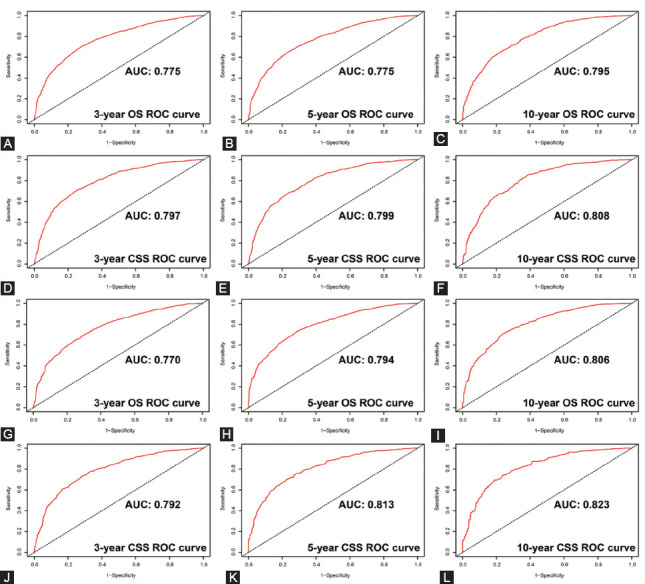
Receiver operating characteristic (ROC) curves predicting the 3-, 5- and 10-year overall survival (OS) and cancer-specific survival (CSS) in the training and validation sets. A, 3-year OS rates in the training set; B, 5-year OS rates in the training set; C, 10-year OS rates in the training set; D, 3-year CSS rates in the training set; E, 5-year CSS rates in the training set; F, 10-year CSS rates in the training set; G, 3-year OS rates in the validation set; H, 5-year OS rates in the validation set; I, 10-year OS rates in the validation set; J, 3-year CSS rates in the validation set; K, 5-year CSS rates in the validation set; L, 10-year CSS rates in the validation set.

**FIGURE 5:**
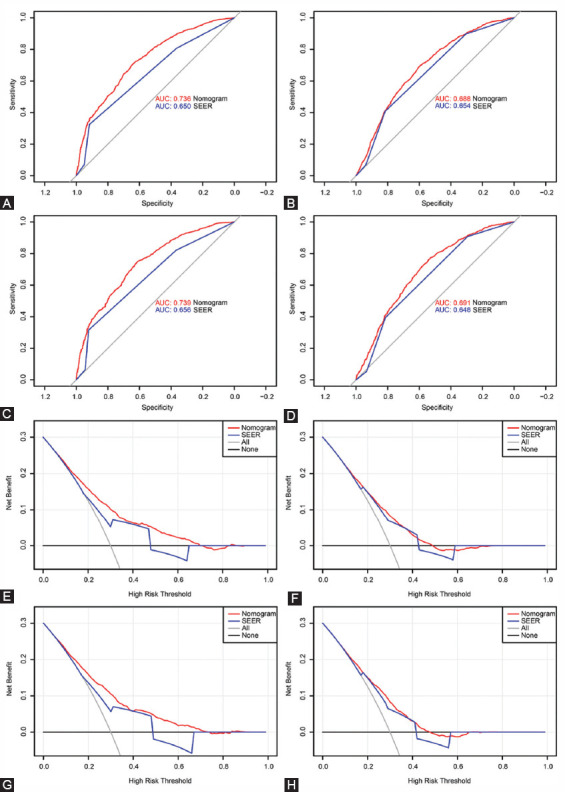
Receiver operating characteristic (ROC) curves and decision curve analysis (DCA) curves detects the predictive value of the nomograms in the training and validation sets. A. ROC curve for overall survival (OS) in the training set; B. ROC curve for cancer-specific survival (CSS) in the training set; C. ROC curve for OS in the validation set. D. ROC curve for CSS in the validation set; E. DCA curve for OS in the training set; F. DCA curve for CSS in the training set; G. DCA curve for OS in the validation set. H. DCA curve for CSS in the validation set.

**FIGURE 6:**
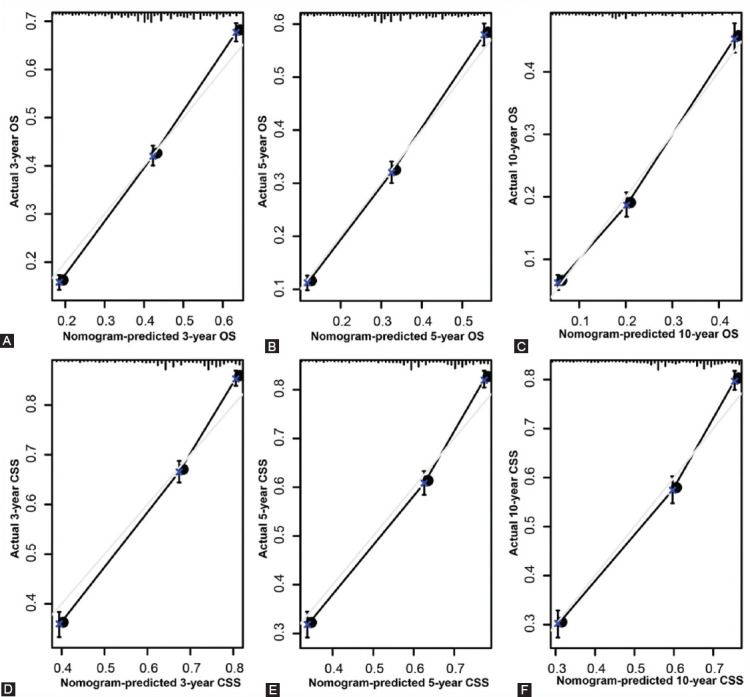
Calibration plot of the nomogram for predicting 3-, 5-, and 10-year overall survival (OS) and cancer-specific survival (CSS) in the training set. A. 3-year OS; B. 5-year OS; C. 10-year OS; D. 3-year CSS; E. 5-year CSS; F. 10-year CSS.

**FIGURE S2:**
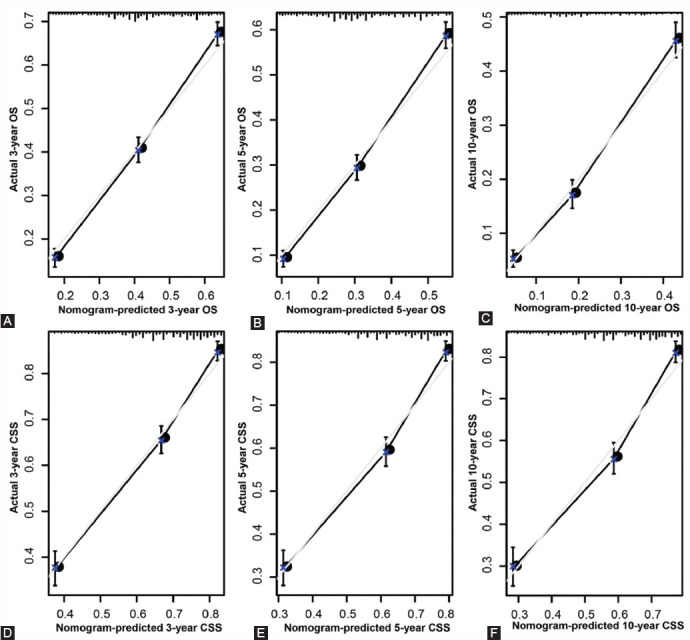
Calibration plot of the nomogram for predicting 3-, 5-, and 10-year overall survival (OS) and cancer-specific survival (CSS) in the validation set. A. 3-year OS; B. 5-year OS; C. 10-year OS; D. 3-year CSS; E. 5-year CSS; F. 10-year CSS.

We evaluated the predictive performance of the nomogram for 3-, 5- and 10-year OS and CSS in the training and validation cohorts and found that the nomograms provided a good assessment of OS and CSS at 3, 5 and 10 years in UTUC patients (Figure 4). In the training set, whether predicting OS or CSS, time-dependent ROC curves (OS: nomogram: AUC=0.736, 95% CI, 0.723-0.748; SEER stage: AUC=0.650, 95% CI, 0.637-0.664; p<0.001; CSS: nomogram: AUC=0.688, 95% CI, 0.677-0.698; SEER stage: AUC=0.654, 95% CI, 0.643-0.665; p<0.001) and C-index (OS: nomogram: C-index=0.701, 95% CI, 0.693-0.709; SEER stage: C-index=0.656, 95% CI, 0.648-0.664; p<0.001; CSS: nomogram: C-index=0.729, 95% CI, 0.719-0.739; SEER stage: C-index=0.685, 95% CI, 0.675-0.695; p<0.001) showed high accuracy of the nomograms compared to SEER stage (Figure 5A-D). In addition, DCA curves also showed better clinical utility of the nomogram (Figure 5E-H). Moreover, we calibrated the 3-, 5- and 10-year OS and CSS nomograms of the entire cohort. The results showed good consistency between the prediction of the nomogram and the actual observation (Figure 6 and Supplementary Figure 2). The results indicate that the model established by the nomogram was quite accurate.

## DISCUSSION

UTUC is a relatively rare solid tumor, accounting for 5-7% of all kidney tumors and 5-10% of all urothelial tumors.[13] However, 60% of UTUC cases are invasive at the time of diagnosis, 15-25% are associated with bladder tumors, 7% have metastasis, and the 5-year CSS rate is approximately 50-80%.[13, 14] Therefore, it is still very important to understand the incidence trend of UTUC and to build a model that can accurately predict the prognosis of UTUC patients.

In this approximately 30-year retrospective study, we first examined the incidence trend of UTUC. The results showed that the average age at onset of UTUC increased from 70.01 years in 1988 to 73.20 years in 2015. The mean age of onset of UTUC in females and the ureter was higher than that in males and the renal pelvis. Regarding the incidence of UTUC, we found that the incidence of UTUC decreased from 1.55/100,000 persons in 1988 to 1.52/100,000 persons (APC=-0.1). In the same period, the incidences of UTUC in males, patients 70+ years old and the renal pelvis were higher than those in females, patients <60 years or 60-69 years old and patients with UTUC in the ureter. Then, we constructed a reliable and accurate nomogram based on important clinical factors to predict OS and CSS in patients with UTUC.

Previous studies have also studied changes in the incidence of UTUC. Munoz et al.[15] assessed the changes in the incidence of UTUC from 1973 to 1996 with reference to the 1980 United States census and concluded that the incidence of ureteral tumors seemed to have increased slightly (0.69 to 0.73/100,000 person-years). Through the study of UTUC patients from 1973 to 2005, Raman et al.[16] found that the overall incidence of UTUC increased slowly (from 1.88 to 2.06 per 100,000 persons).

Our study found that the overall incidence of UTUC decreased slightly between 1988 and 2015, which may be because Munoz et al. used the 1980 U.S. population (21,526,796) as the standard population, while we used the 2000 U.S. population (26,787,544) as the standard population, resulting in a lower overall incidence than Munoz et al.[15] We speculate that the difference between our results and Raman et al.[16] was due to the impact of the different databases used.

Some studies have pointed out that the male-to-female ratio of UTUC is approximately 2:1 in American or European patients, and a primary site in the renal pelvis is twice as common as in the ureter.[17, 18] Our results are consistent with previous studies. In addition, we also found that in the same period, the incidence of UTUC in patients over 70 years old was significantly higher than that of patients <60 years and 60-69 years. The incidence of UTUC patients aged <60 years (APC=-2.1, 95% CI: -2.3 to -1.9) and 60-69 years (APC=-1.4, 95% CI: -1.5 to -1.2) was decreasing, while the incidence of 70+ years patients was increasing (APC=0.7, 95% CI: 0.6 to 0.8).

The TNM staging and classification system is the basis of the prognosis of patients with UTUC.[19] In this classification system, clinicians can determine the TNM stage according to the depth of tumor invasion (T), lymph node metastasis number (N) and distant metastasis (M), so that clinicians can make personalized treatment plans for patients and evaluate the prognosis of patients. How to better combine the tumor characteristics of patients with their own clinical factors and tailor-made patient risk assessment methods has always been a challenge for clinicians.[20]

The nomogram is a visual tool for predicting prognosis based on multiple variables.[21] The model integrates a variety of prognostic factors and can provide more information for evaluating the survival possibility of individual patients.[22] At present, many cancer nomograms have been developed and show a more accurate prediction of cancer prognosis than traditional TNM systems.[23] In addition, the line chart enables clinicians to include more prognostic factors for patients, to assess patients’ physical condition more intuitively, and to specify personalized treatment plans. Therefore, the establishment of an effective and reliable map is of great significance to the prognosis of patients with UTUC and to provide them with personalized treatment.

Nomograms have been widely used in various malignant tumors.[24-26] Kattan et al. [27] constructed a nomogram that included pretreatment serum prostate-specific antigen levels, biopsy Gleason scores and clinical stages and found that it could predict the 5-year treatment failure probability of clinically localized prostate cancer patients who underwent radical prostatectomy. Similarly, Karakiewicz et al.[28] performed preoperative prediction of 726 patients treated with radical cystectomy and bilateral pelvic lymphadenectomy and found that the multivariate nomogram was more accurate than the TUR T stage alone prediction.

At present, some studies have constructed nomograms of UTUC prognosis.[29-31] Through the study of 227 patients who underwent radical nephroureterectomy, Zeng et al.[30] found that the nomogram based on grade, stage, age, lymph node, concurrent bladder cancer, primary site, histological type and lymphovascular invasion can accurately predict the CSS of UTUC. Krabbe et al.[31] developed a prognostic nomogram composed of four variables, pT stage, pN stage, age and architecture, to predict the relapse-free survival of patients with high-grade UTUC after extirpative surgery. Zhang et al.[29] constructed a nomogram with stronger predictive power than the TNM staging system and SEER stage through 4,990 patients treated with surgery in the SEER database. The subjects of the above studies were UTUC patients who underwent surgery, and the researchers included fewer other prognostic variables. In our study, we developed a nomogram based on thirteen variables of age at diagnosis, sex, marital status, race, origin, primary site, histological type, grade, SEER stage, surgery, radiotherapy and chemotherapy and showed a better ability to predict prognosis than the TNM stage nomogram. Using this nomogram, urologists can evaluate the survival prognosis of patients with UTUC, enabling personalized treatment and monitoring of possible outcomes.

Our research still has some limitations. First, our study is a retrospective study with inevitable selection bias. Second, we could not obtain specific information about radiotherapy and chemotherapy or information about the patient’s physical condition and complications. Moreover, since 15-25% of UTUC patients had bladder cancer, the data of this study did not consider simultaneous or heterochronous bladder cancer.

## CONCLUSIONS

The incidence of UTUC has generally declined in the past 30 years, but it has increased in patients aged 70+ years. Moreover, the prognostic nomogram we established can provide a personalized risk assessment for the survival of UTUC patients.

## Figures and Tables

**Supplemental Table 1:**
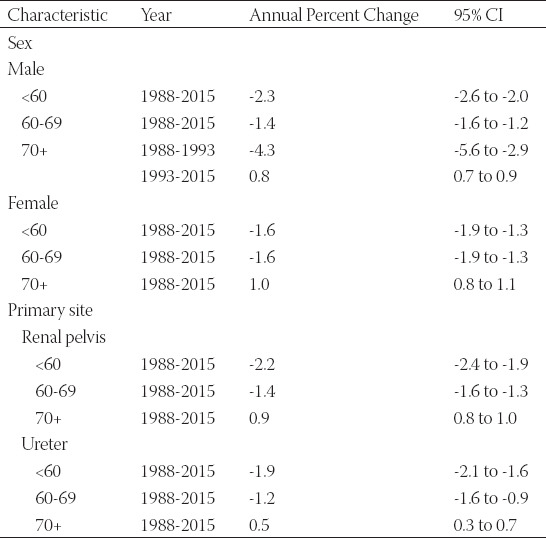
Stratified analysis of changes in the incidence of upper tract urothelial carcinoma (UTUC) patients from 1988 to 2015
